# Effects of Continuous Cropping of Sweet Potato on the Fungal Community Structure in Rhizospheric Soil

**DOI:** 10.3389/fmicb.2019.02269

**Published:** 2019-10-02

**Authors:** Zhiyuan Gao, Meikun Han, Yaya Hu, Ziqian Li, Chaofang Liu, Xue Wang, Qing Tian, Weijing Jiao, Jianmin Hu, Lanfu Liu, Zhengjun Guan, Zhimin Ma

**Affiliations:** ^1^The Laboratory of Sweet Potato, Institute of Cereal and Oil Crops, Hebei Academy of Agriculture and Forestry Sciences, Shijiazhuang, China; ^2^The Key Laboratory of Crop Genetics and Breeding of Hebei, Shijiazhuang, China; ^3^Agricultural Product Quality Inspection Center of Shijiazhuang, Shijiazhuang, China; ^4^Department of Life Science, Yuncheng University, Yuncheng, China

**Keywords:** sweet potato, continuous cropping, Illumina Miseq method, rhizospheric soil, fungal, community structure

## Abstract

Soil microorganisms play an important role in the ecosystem, and have a certain relationship with the continuous cropping obstacles, which are common with sweet potato. However, there are few reports on the effects of continuous cropping of sweet potato on the microbial community structure in the rhizospheric soil. Here, we investigated the effects of continuous cropping of sweet potato on the fungal community structure in rhizospheric soil, in order to provide theoretical basis for prevention and control of continuous cropping obstacles. This study used X18 and Y138 varieties as experimental materials. Soil samples were collected during the early period of planting and harvest in two consecutive years, and fungi were analyzed using Illumina Miseq. Results showed that the fungi diversity and richness in rhizospheric soil of X18 and Y138 were significantly increased after continuous cropping; the most dominant fungi phylum was Ascomycota, which decreased significantly after continuous cropping. In addition, the content of beneficial fungi such as *Chaetomium* was reduced, while that of harmful fungi such as *Verticillium*, *Fusarium*, and *Colletotrichum* were increased. The composition of X18 and Y138 fungal community in the same sampling period after continuous cropping was similar, although that of the same sweet potato variety significantly differed with the sampling period. Overall, our results indicate that continuous cropping alters the fungal community structure of the sweet potato rhizospheric soil, such that the content of beneficial fungi decrease, while that of harmful fungi increase, thereby increasing soil-borne diseases and reducing the yield and quality of sweet potato. Furthermore, these effects are different for different sweet potato varieties. Thus, during actual production, attention should be paid to maintain the stability of sweet potato rhizospheric soil micro-ecology through rotation or application of microbial fertilizers and soil amendments to alleviate continuous cropping obstacles.

## Introduction

Sweet potato [*Ipomoea batatas* (L.) Lam.] is an important global food, feed, and industrial raw material. It is a high-yield and high-efficiency crop that is drought-tolerant and resistant to ridges, exhibiting strong adaptability. The cultivation area and yield of sweet potato are second only to rice, wheat, and corn (Song and Wang, 2007; Dai et al., 2015). Recently, with the adjustment of planting structures in China and the implementation of underground water pressure mining and other policies in North China, the planting area of sweet potato has increased yearly, and large-scale intensive production has accelerated. However, continuous cropping obstacles are common with sweet potato. Studies have shown that the continuous cropping of sweet potato can cause a 20–30% reduction in yield, as well as severe plant death or even lack of production (Zhou and Ma, 2003; Jia et al., 2010). Due to the limited arable land in China, the area used for continuous cropping of sweet potato or multiple cropping is getting larger, and thus the continuous cropping obstacle could become a bottleneck problem, restricting the sustainable development of the sweet potato industry in China.

Soil microorganisms play an important role in the ecosystem (Sun et al., 2015) and are the key factors associated with soil quality (Beckers et al., 2017), soil fertility, and productivity (Blagodatskaya and Kuzyakov, 2013). Changes in rhizospheric soil microorganisms affect the absorption and transformation of soil nutrients (Fierer et al., 2012). Accordingly, the quantity and species of rhizospheric soil microorganisms are important factors that affect the growth, development, and health status of plants (Berendsen et al., 2012; Bron et al., 2012; Jin, 2012). Numerous studies have shown that continuous cropping affects the rhizospheric soil microbial structure (Huang et al., 2013; Qiao et al., 2013; Guo et al., 2017; Sun et al., 2017). In turn, such alterations further contribute to the aggravation of continuous cropping obstacles (Sun, 2017). Therefore, the relationship between microbial community structure in the rhizospheric soil and continuous cropping obstacles has attracted increasing attention.

Fungi are important decomposers in soil, and the composition and diversity of their community structure play an important role in the balance of the ecosystem (Xu et al., 2015). In addition, many fungi are closely related to plant diseases (Shang, 2013). Studies have shown that under continuous cropping conditions, soil-borne diseases of sweet potato are aggravated. For example, root rot is a devastating soil-borne disease of sweet potato caused by *Fusarium*, which seriously affects the yield and quality of sweet potato (Li et al., 2009). In contrast, some fungi can also be beneficial by inhibiting pathogenic bacteria (Tan et al., 2017). Thus, there is a close relationship between continuous cropping obstacles and the structure of fungal communities, which has attracted wide attention at home and abroad.

The culture-independent Illumina Miseq high-throughput sequencing technology can be used to directly identify microbial communities and detect changes in species with low abundance. At present, this technology has been widely applied to study the microbial diversity in waste-water, soil, and the human gut (Lisa et al., 2013; Bai et al., 2015).

Li et al. (2018) applied high-throughput sequencing technology to study the effects of continuous cropping on the fungal community structure in rhizospheric soil of outdoor strawberry. The results showed that the key beneficial fungi decreased dramatically, and a number of pathogenic fungi increased significantly, which significantly contributed to the continuous cropping obstacle in case outdoor strawberry. Liu et al. (2019a) studied the response of soil fungal community structure to long-term continuous cropping of soybean, which showed that the abundance of soil fungi after long-term continuous cropping was significantly higher than that after 2 years of continuous cropping and rotation. Thus, long-term continuous cropping can change the composition of fungi.

Although several studies have focused on the prevention and control of pests and diseases of sweet potato (Yin, 2015; Johnson and Gurr, 2016), only few reports exist on the effects of continuous cropping of sweet potato on the microbial community structure in the rhizospheric soil. So far, there is no report on the effects of continuous cropping of sweet potato on fungal community structure in rhizospheric soil using high-throughput method. Therefore, in this study, we applied the Illumina Miseq high-throughput sequencing technology to analyze changes in fungal community structure in the rhizospheric soil of different sweet potato varieties in different continuous cropping years, and explored the effects of continuous cropping of sweet potato on the fungal community structure. The aim of this study is to provide a theoretical basis for alleviating the continuous cropping obstacles of sweet potato and study the mechanism of continuous cropping obstacles and biological control of sweet potato root rot, which has important practical significance to guide agricultural practices.

## Materials and Methods

### Description of the Study Area and Materials

The experimental site was located in the Dishang test station of Institute of Cereal and Oil Grops, Hebei Academy of Agriculture and Forestry Sciences at Shijiazhuang, Hebei Province, China (37°56′24.62″ N, 114°42′46.96″ E). This region has an average temperature of 12.5°C and a mean annual precipitation of 494 mm. The pH of the tested soil was 8.40, the available nitrogen was 85.83 mg kg^−1^, available phosphorus was 24.08 mg kg^−1^, and available potassium was 114.17 mg kg^−1^. We used Xushu 18 (X18, resistant to continuous cropping) and Yizi 138 (Y138, susceptible to continuous cropping) as experimental materials. And X18 is mostly used for starch processing. Y138 is a variety that people like to eat for its high sugar content. Both are important sweet potato varieties, and have high market demand.

### Experimental Design and Sample Collection

The experiment began in 2015. Sweet potato seedlings were planted on May 6 and harvested on October 7 each year, and planted for two consecutive years. We adopted a random block design, with three experimental repetitions. The ridge distance was 85 cm, seedling spacing was 25 cm for five rows, length of the plot was 6 m, and the area was 25.5 m^2^, and the planting management was the same as for the field. Rhizospheric soil was collected 30 days after planting and 7 days before harvest, respectively. According to the sampling method of Sun et al. (2009), the samples were taken in the “S” shape. The whole root of the sweet potato was completely excavated with a sampling shovel and then the root was tapped; the naturally dropped soil that was loosely adhered to the root was discarded, and the soil closely adhered to the root was collected. The sample was passed through a 2-mm sieve and stored at −80°C for microbial analysis.

### DNA Extraction, PCR Amplification, and Illumina MiSeq Sequencing

Microbial DNA was extracted from the samples using the E.Z.N.A.^®^ Soil DNA Kit (Omega Bio-tek, Norcross, GA, U.S.) according to the manufacturer’s protocols. The fungal 18S rRNA gene was amplified by PCR (95°C for 2 min, followed by 25 cycles at 95°C for 30 s, 55°C for 30 s, and 72°C for 30 s and a final extension at 72°C for 5 min) using primers 817F 5′-barcode-817F TTAGCATGGAATAATRRAATAGGA)-3′ and 1196R 5′-TCTGGACCTGGTGAGTTTCC-3′, where barcode was an eight-base sequence unique to each sample. PCR reactions and the extraction and purification of amplicons were performed according to the report by Yang et al. (2018a). Purified PCR products were quantified using Qubit^®^3.0 (Life Invitrogen) and every 24 amplicons whose barcodes were different were mixed equally. The pooled DNA product was used to construct Illumina Pair-End library following Illumina’s genomic DNA library preparation procedure. Then the amplicon library was paired-end sequenced (2 × 250) on an Illumina MiSeq platform (Shanghai BIOZERON Co., Ltd.) according to the standard protocols.

### Processing and Analysis of the Sequencing Data

High-quality sequences were obtained by quality control and filtering of sequence quality as described by Yang et al. (2018a). Operational Taxonomic Units (OTUs) were clustered with 97% similarity cutoff using UPARSE (version 7.1 http://drive5.com/uparse/) and chimeric sequences were identified and removed using UCHIME. The phylogenetic affiliation of each 18S rRNA gene sequence was analyzed by RDP Classifier^1^ against the Unite (Release 6.0) 18S rRNA database using a confidence threshold of 70% (Yang et al., 2018a).

The richness and diversity indexes were generated based on Mothur v.1.21.1 (Schloss et al., 2009), including the Ace, Chao, Shannon, and Coverage. The principal coordinate analysis (PCoA) was conducted using the community ecology package, R-forge (Vegan 2.0 package). Venn diagram was prepared using VennDiagram to analyze overlapped and unique OTUs during the treatment processes. Heatmap figures were generated using the Vegan packages in R to analyze the composition of fungal community.

## Results

### α-Diversity of Fungal in the Rhizospheric Soil of Sweet Potato

**Figure 1:**
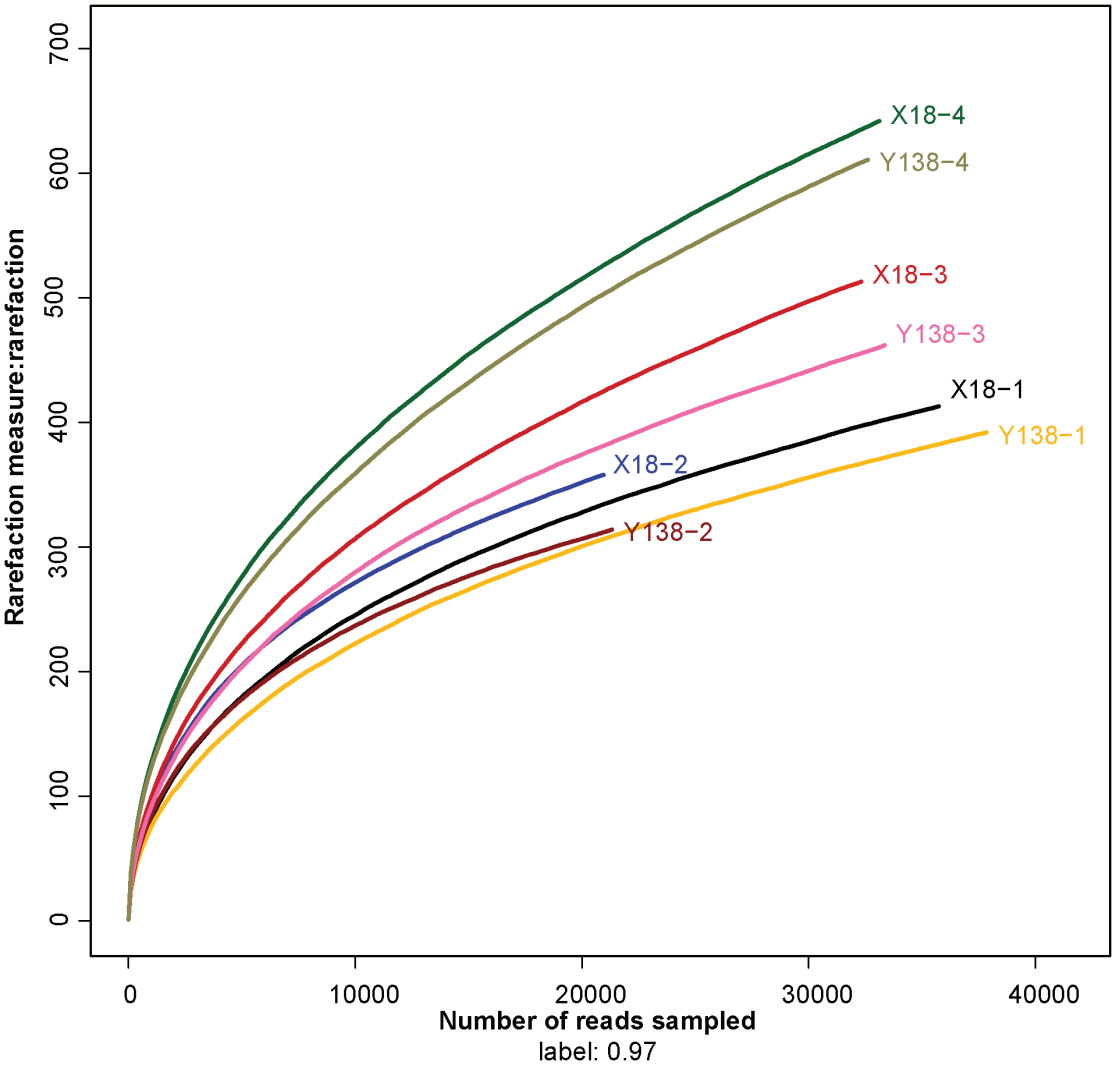
Rarefaction curves for all soil samples. X18, Xushu 18; Y138, Yizi 138; 1 and 2 represent sampling of early planting and early harvest in 2015, 3 and 4 represent sampling of early planting and early harvest in 2016, respectively.

The collected samples were subjected to high-throughput sequencing using the MiSeq platform, and the sequencing results are shown in Table 1. The coverage of all samples were above 99.16%, and the rarefaction curve of each samples had already approached a saturation plateau (Figure 1), which indicated that the sequencing library had reached saturation, and the results can truly reflect the sample condition. The reads per sample ranged from 20,955 to 37,849. The OTU per sample ranged from 314 to 642. With an increase of continuous cropping years, the OTU of rhizospheric soil fungi showed an overall increasing trend during the sampling period of X18 and Y138, the Ace and Chao also showed an increasing trend, indicating that the richness of X18 and Y138 rhizospheric soil fungi increased with increase in the continuous cropping years. The Shannon index also showed an increasing trend, indicating that the diversity of fungi of sweet potato rhizospheric soil increased after continuous cropping. Notably, the Ace, Chao, and Shannon indices of X18 were higher than those of Y138 after continuous cropping, indicating that X18 had higher fungal richness and diversity than Y138.

**Table 1:** MiSeq sequencing results and α-diversity index of sweetpotato rhizospheric soil samples.

Sample ID	Reads	0.97				
		OTU	Ace	Chao	Coverage	Shannon
X18-1	35,742	413	771 (701, 858)	643 (564, 763)	0.995495	3.12 (3.1, 3.14)
X18-2	20,955	358	600 (547, 669)	547 (477, 659)	0.993749	3.2 (3.17, 3.22)
X18-3	32,317	513	1,087 (991, 1,201)	899 (779, 1,072)	0.993161	3.11 (3.08, 3.13)
X18-4	33,117	642	1,400 (1,289, 1,530)	1,132 (993, 1,325)	0.991636	3.77 (3.75, 3.79)
Y138-1	37,849	392	729 (660, 816)	641 (555, 774)	0.995773	2.86 (2.84, 2.88)
Y138-2	21,328	314	450 (406, 515)	476 (413, 581)	0.994702	3 (2.97, 3.02)
Y138-3	33,351	462	878 (802, 971)	788 (680, 948)	0.994363	2.88 (2.86, 2.9)
Y138-4	32,619	611	1,415 (1,298, 1,553)	1,083 (948, 1,271)	0.991723	3.75 (3.73, 3.77)

*X18, Xushu 18; Y138, Yizi 138; 1 and 2 represent sampling of early planting and early harvest in 2015, 3 and 4 represent sampling of early planting and early harvest in 2016, respectively; Reads: the optimized sequences; OTU, operational taxonomic unit. The numbers within parentheses are the lower and upper limits in statistics of the corresponding data, respectively*.

### Community Composition of Fungal in the Rhizospheric Soil of Sweet Potato

Analysis of fungal community structure showed that the fungus diversity of sweet potato rhizospheric soil increased significantly after continuous cropping. Additionally, the fungal content of rhizospheric soil was significantly different between X18 and Y138 in different continuous cropping periods.

**Figure 2:**
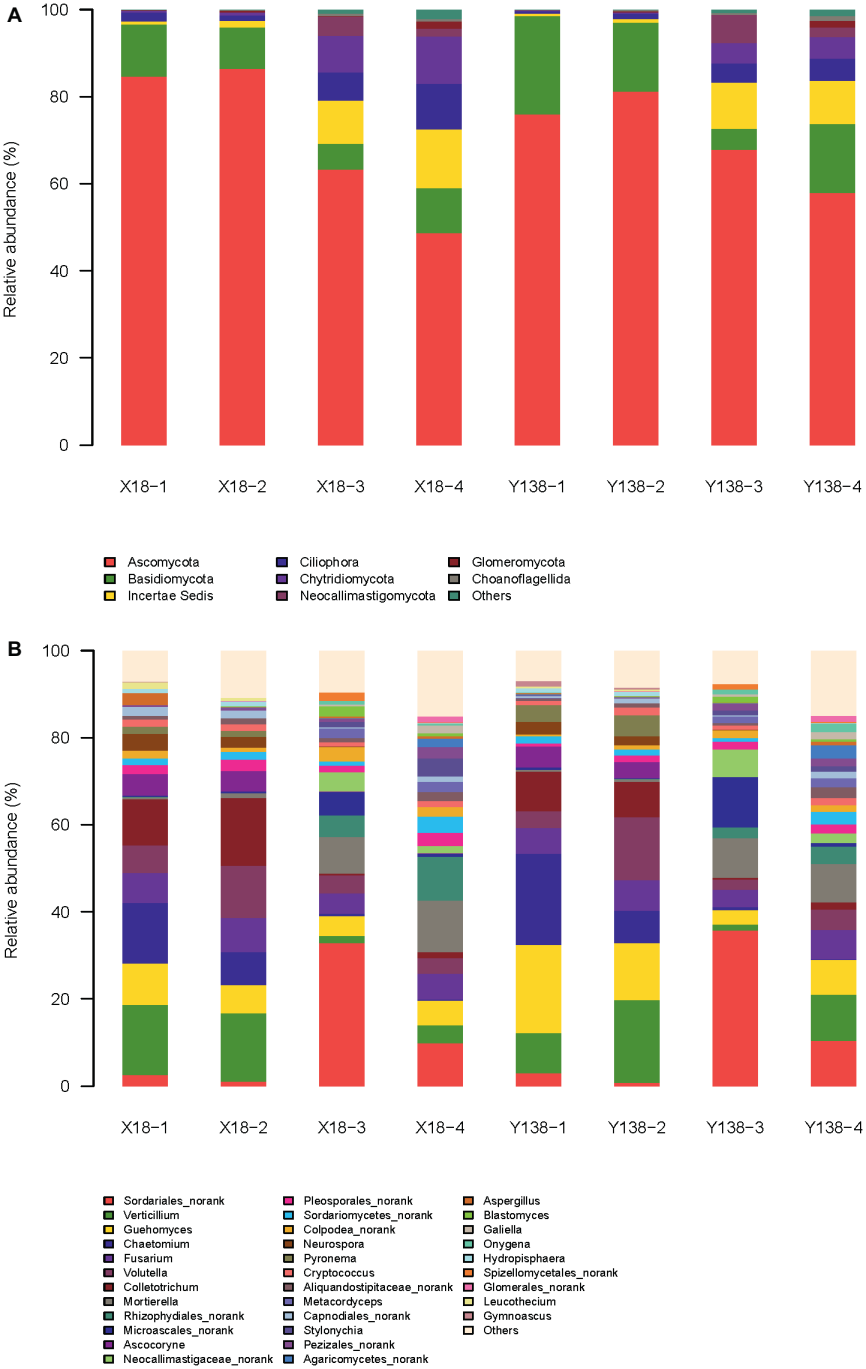
Relative abundance of the fungal phyla **(A)** and genera **(B)** in the rhizospheric soil of continuous cropping sweet potato X18 and Y138; X18, Xushu 18; Y138, Yizi 138; 1 and 2 represent sampling of early planting and early harvest in 2015, 3 and 4 represent sampling of early planting and early harvest in 2016, respectively.

At the phylum level (Figure 2A), Ascomycota, Basidiomycota, Incertae Sedis, and Chytridiomycota were the dominant phyla in the X18 and Y138 rhizospheric soil. Among them, Ascomycota was the most abundant, accounting for 84.6%, which decreased significantly after continuous cropping (*p* < 0.05). Moreover, continuous cropping had a greater effect on the Ascomycota content of X18 rhizospheric soil than that of Y138. After continuous cropping for 2 years, the content of Ascomycota of X18 decreased by 21.37 and 37.77%, respectively at the early stage of planting and harvest, and Y138 decreased by 8.13 and 23.28%, respectively. (*p* < 0.05). The content of Basidiomycota was second to Ascomycota, and also decreased with increase in continuous cropping time; slight increase was observed in the early harvest of 2016. The content of Incertae Sedis and Chytridiomycota increased with continuous cropping of sweet potato. The increment of both was more in X18 rhizospheric soil than in Y138 by 1.4 and 2.3 times of Y138, respectively. Blastocladiomycota is one of the nondominant fungi. After continuous cropping for 2 years, the content of Blastocladiomycota of X18 increased by 0.40 and 0.33% in the early stage of planting and harvest, respectively, and Y138 increased by 0.40 and 0.14%, respectively.

Some protozoa such as Ciliophora were also detected in the rhizospheric soil of sweet potato.

At the genus level (Figure 2B), the dominant genus of the X18 and Y138 rhizospheric soil were *Verticillium*, *Chaetomium*, *Colletotrichum*, *Guehomyces*, *Fusarium*, *Volutella*, and *Sordariales_norank*.

*Verticillium*, *Colletotrichum*, *Fusarium*, and *Guehomyces* showed a tendency of first decreasing and then increasing with the increase in continuous cropping time. The former three are important plant pathogens (Li et al., 2009; Jing et al., 2018; Liu et al. 2019b), and had a negative effect on the growth of sweet potato.

*Chaetomium* shows potential biocontrol effect on many plant pathogens (Huang, 2009). With the increase in continuous cropping years, *Chaetomium* content gradually decreased, indicating that continuous cropping reduces the content of beneficial microorganisms. Notably, its reduction in Y138 was about 1.6 times that in X18 (*p* < 0.05).

The content of *Sordariales*_*norank* in rhizospheric soil of X18 and Y138 was not significantly different, with more content in the early stage of planting and less in the early stage of harvest. Moreover, the content of *Sordariales_norank* in each period of 2016 was significantly increased compared with that in each period of 2015 (*p* < 0.05), and the content was the highest in the early stage of planting in 2016, accounting for 32.9 and 35.8%, respectively in X18 and Y138.

### Heatmap Analysis of Fungal in the Rhizospheric Soil of Sweet Potato

**Figure 3:**
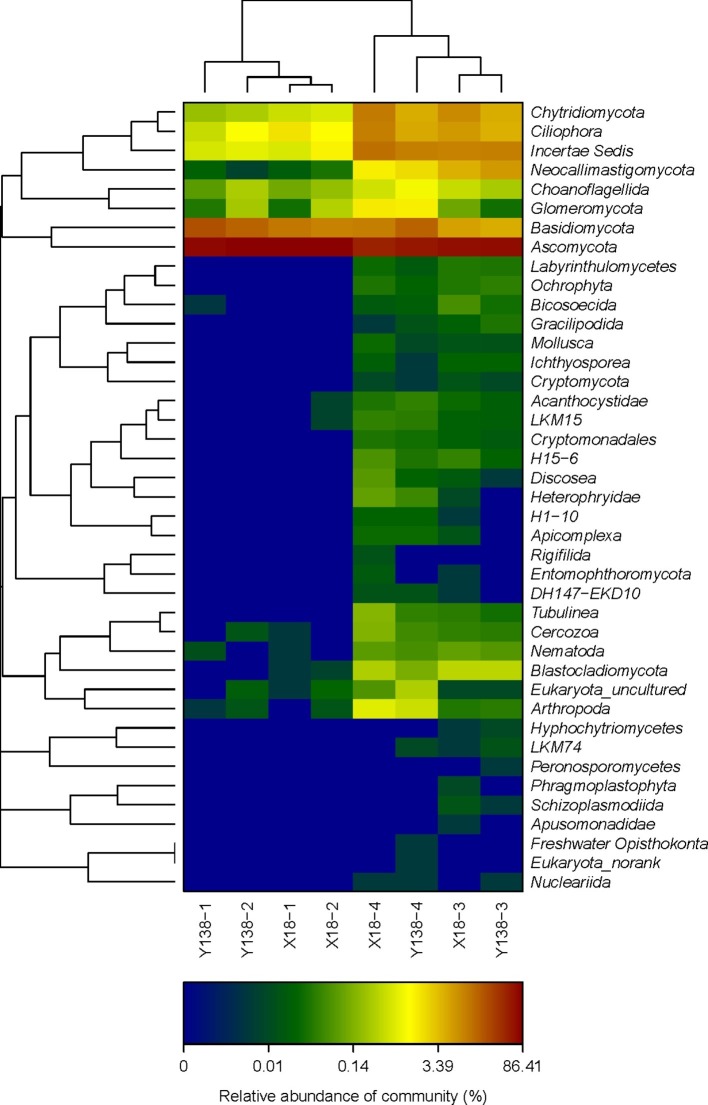
Microbial community heatmap analysis of the fungal phyla detected across all samples. X18, Xushu 18; Y138, Yizi 138; 1 and 2 represent sampling of early planting and early harvest in 2015, 3 and 4 represent sampling of early planting and early harvest in 2016, respectively. The relative values for fungal phyla are indicated by color intensity with the legend at the bottom of the picture.

The changes of fungal community structure in the rhizospheric soil of X18 and Y138 after continuous cropping can be seen more clearly from the relative scale value and color change of heatmap (Figure 3), At the phylum level, the fungal microbial species in X18 and Y138 rhizospheric soil were less and the species diversity was lower in 2015. After continuous cropping, the fungal diversity of X18 and Y138 rhizospheric soil increased. Furthermore, it can be seen from the sample cluster analysis of the heatmap that all the samples were clustered into two groups and the samples of the same continuous cropping year were grouped together.

### Venn Analysis of Fungal in the Rhizospheric Soil of Sweet Potato

**Figure 4:**
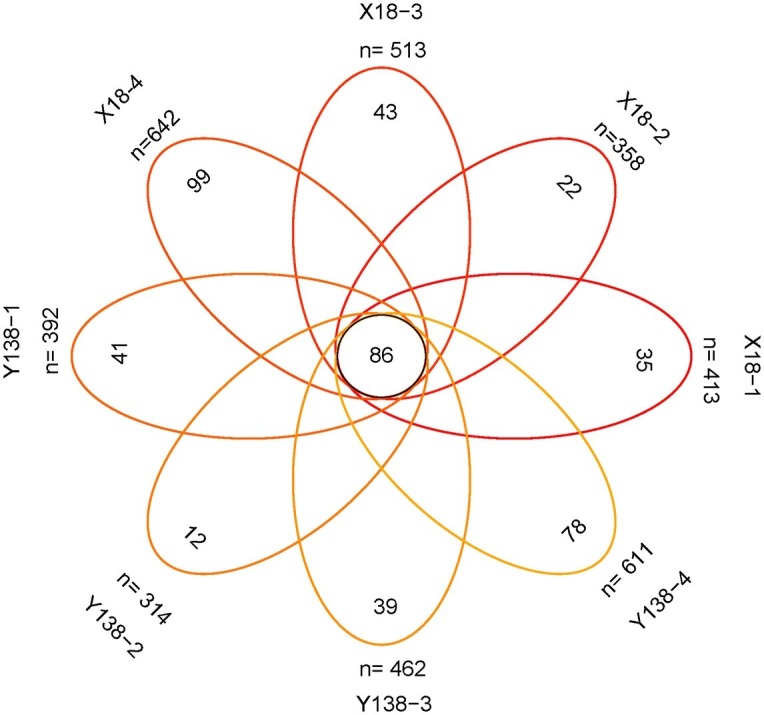
Venn analysis of the number of common and unique operational taxonomic unit (OTU). X18, Xushu 18; Y138, Yizi 138; 1 and 2 represent sampling of early planting and early harvest in 2015, 3 and 4 represent sampling of early planting and early harvest in 2016, respectively.

The number of fungal OTUs shared by X18 and Y138 samples for each sampling period was 86 (Figure 4). In the continuous cropping period, the unique OTUs in each sampling period were relatively small. In the four samples of X18 and Y138, the unique OTUs were 35, 22, 43, and 99 and 41, 12, 39, and 78, respectively, showing a trend of decreasing first and then increasing, which was consistent with the changing trend of the total OTUs. The results showed that continuous cropping caused changes in the fungal community structure of sweet potato rhizospheric soil.

### PCoA and Cluster Analysis of Fungal in the Rhizospheric Soil of Sweet Potato

**Figure 5:**
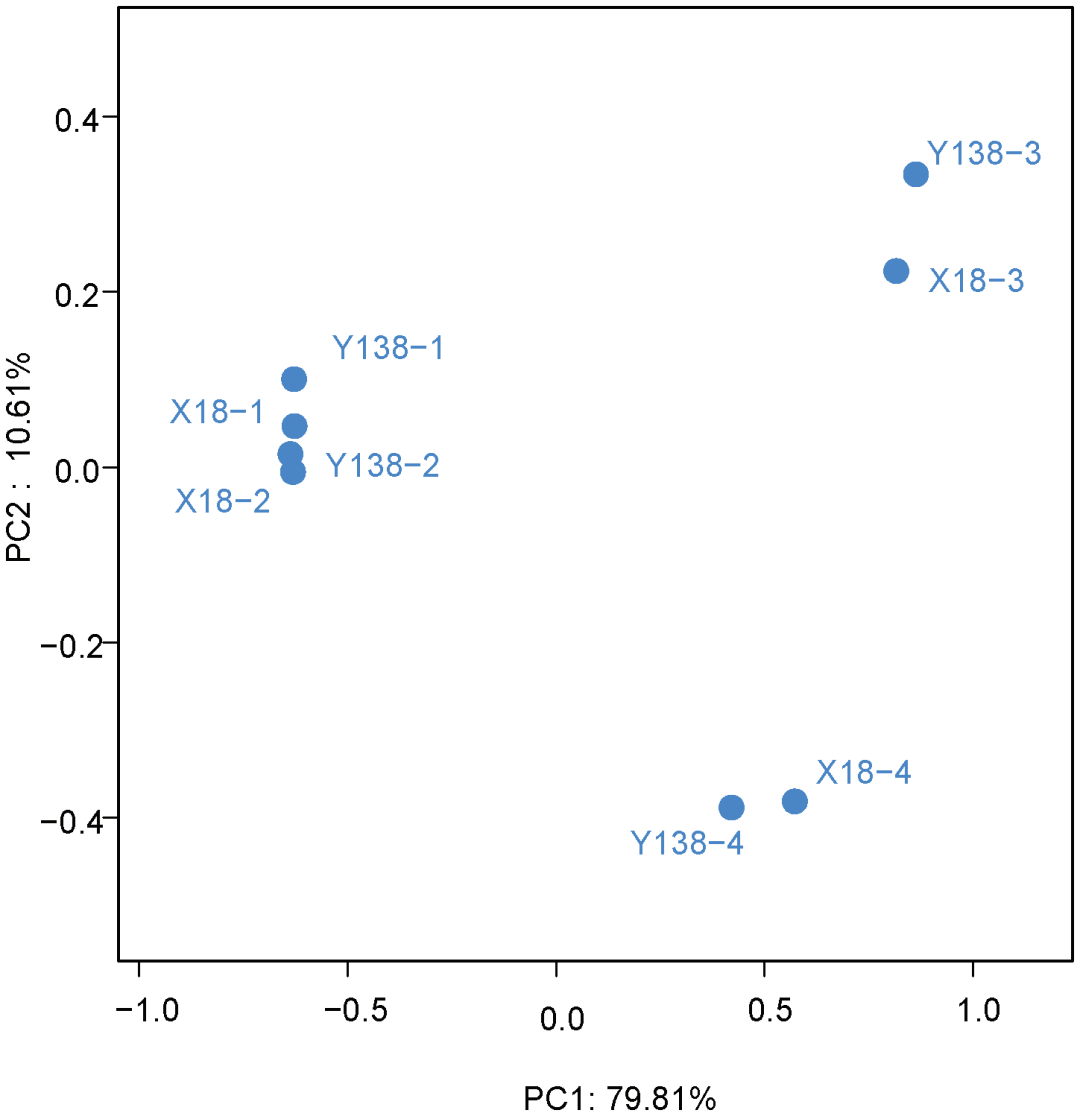
Principal coordinate analysis of operational taxonomic units. X18, Xushu 18; Y138, Yizi 138; 1 and 2 represent sampling of early planting and early harvest in 2015, 3 and 4 represent sampling of early planting and early harvest in 2016, respectively.

We next carried out the PCoA of X18 and Y138 rhizospheric soil fungi (Figure 5). The two main coordinates extracted explained 90.42% of the variation, of which PC1 explained 79.81% of the variation; PC2 explained 10.61% of the variation. It showed that the distribution of X18 samples at different sampling times was relatively discrete and the samples moved further apart. A similar trend was observed for Y138, indicating that the fungal community structure of rhizospheric soil of X18 and Y138 changed with the extension of continuous cropping time. However, the distance among different sweet potato varieties with the same sampling time were close, consistent with the cluster analysis results, which indicated that the structure of fungal community was similar in the same sampling period. Meantime, the samples in 2015 were close together, while the samples in 2016 were far apart, indicating that there were little differences in fungal community structure in the samples in 2015, while the effects on the fungal community structure of sweet potato rhizospheric soil was larger with increase in the continuous cropping years.

**Figure 6:**
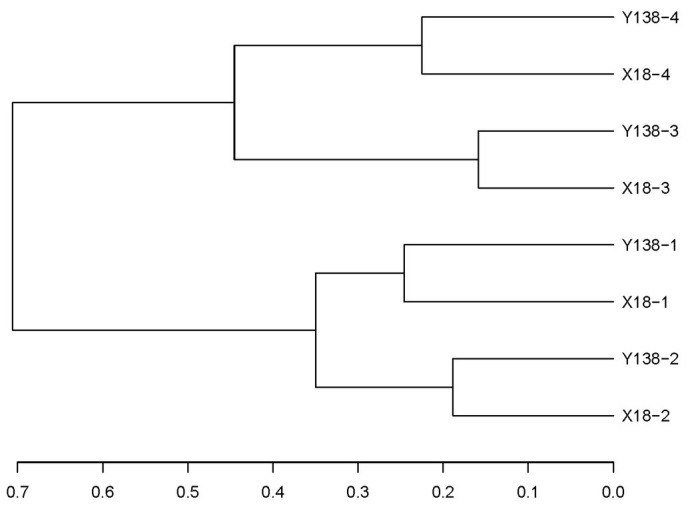
Results of Unifrac cluster analysis of operational taxonomic units in the rhizospheric soil of X18 and Y138 at different sampling time. X18, Xushu 18; Y138, Yizi 138; 1 and 2 represent sampling of early planting and early harvest in 2015, 3 and 4 represent sampling of early planting and early harvest in 2016, respectively.

In the cluster analysis (Figure 6), all samples clustered into two large groups, among which the samples of the same continuous cropping years clustered into one group, respectively. This indicated that the continuous cropping time had certain influence on the fungal community in the rhizospheric soil of sweet potato, and the structures of the fungal community of X18 and Y138 rhizosphere soils with the same continuous cropping time were similar, while that of with different continuous cropping years were different.

## Discussion

The Illumina Miseq high-throughput technique was used to analyze the fungal community structure of sweet potato rhizospheric soil after continuous cropping. It was found that the number of OTUs and the Chao, Ace, and Shannon indexes increased after continuous cropping, indicating that sweet potato continuous cropping lead to an increase in the diversity and richness of fungi in the rhizospheric soil, which was consistent with the results of previous studies on the changes of fungal community structure in the rhizospheric soil of Panax notoginseng and American ginseng (Dong et al., 2017; Tan et al., 2017). Moreover, our previous study also showed an increase in fungal content of sweet potato rhizospheric soil after continuous cropping by phospholipid fatty acid analysis (Gao et al., 2019).

Tan et al. (2017) found that the dominant phyla in rhizospheric soil fungi after Panax notoginseng continuous cropping were Ascomycota, Zygomycota, Basidiomycota, and Chytridiomycota. Our study found that the dominant phyla of rhizospheric soil fungi after X18 and Y138 continuous cropping were Ascomycota and Basidiomycota, which was consistent with previous studies (Bai et al., 2015; Yang et al., 2018b). With the increase in continuous cropping years, the content of Ascomycota decreased significantly (*p* < 0.05), which was consistent with the results of Feng et al. (2019). Ascomycota plays an important role in the degradation of organic matter in rhizospheric soil (Schoch et al., 2009), and a decrease in its content may impact soil fertility.

The dominant genus in the X18 and Y138 rhizospheric soil were *Verticillium*, *Chaetomium*, *Colletotrichum*, *Guehomyces*, *Fusarium*, *Volutella*, and *Sordariales_norank*. Among them, *Chaetomium* is a beneficial fungus with biocontrol effect (Huang, 2009), and its content was reduced after sweet potato continuous cropping. In contrast, the content of *Verticillium,* which can cause verticillium wilt in plants (Liu et al., 2015; Jing et al., 2018), *Colletotrichum*, another important plant pathogen (Liu et al., 2019b), and *Fusarium*, which is a pathogen that difficult to control in production and causes several plant diseases, including sweet potato root rot (Hu, 1984), were increased with continuous cropping of sweet potato. The increase in the content of pathogenic fungi in the rhizospheric soil can result in an increase in the incidence of sweet potato root rot (Yin et al., 2017), which in turn can promote continuous cropping obstacles. This would then form a vicious circle that seriously affects the production of sweet potato. In the study, we found that the yield of X18 and Y138 decreased significantly after continuous cropping. The average yield of X18 for 2 consecutive years was 12.36 and 10.64 t hm^−2^, respectively. Y138 was and 2.66 t hm^−2^, respectively. The yield reduction of Y138 was significantly higher than that of X18 (*p* < 0.05). Thus, continuous cropping resulted in an imbalance of fungal community structure in the rhizospheric soil of sweet potato, with a reduction in the beneficial fungal content and increase in the content of harmful or pathogenic fungi. These results are consistent with a previous study (Guo et al., 2017). Therefore, the balance of sweet potato rhizospheric soil microecology is crucial for the healthy growth of sweet potato. In actual production, ecological means such as rational application of microbial fertilizer and soil amendments (Qiao, 2011; Tao et al., 2014; Cai et al., 2015) can be used to increase the content of beneficial microorganisms in the soil to alleviate continuous cropping obstacles, as well as prevent and control sweet potato root rot, thereby avoiding the use of large amounts of fertilizers and pesticides that can cause environmental pollution.

Continuous cropping also led to differences in the fungal community composition of the rhizospheric soil among different sweet potato varieties, since the OTU, Ace, Chao and Shannon indexes of X18 after continuous cropping were higher than those of Y138.

Based on cluster analysis and PCoA, it was found that the rhizospheric soil fungus community structure of sweet potato was significantly different in different sampling periods after continuous cropping, but the difference between the two varieties was relatively small in the same sampling period, indicating that the sampling period or season also had some influence on the rhizospheric soil fungus community structure (Li et al., 2017).

The changes of microbial community structure in rhizospheric soil are crucially related to the continuous cropping obstacles of sweet potato. This study provide a theoretical basis for alleviating the continuous cropping obstacles of sweet potato and study the mechanism of continuous cropping obstacles and biological control of sweet potato root rot, and the study has important practical significance to guide agricultural practices. This will also become an important aspect of sweet potato research. Further research on the application of microbial methods, such as the development of special microbial fertilizer for sweet potato, to prevent and control the continuous cropping obstacles of sweet potato is needed.

Continuous cropping obstacles are caused by complex factors within the soil-crop-microbial system, and needs to be solved through a combination of multiple analytical methods. There are many factors related to continuous cropping obstacles, such as soil enzyme activity, soil physical and chemical properties, and root exudates (Sun et al., 2017). Some studies have reported that continuous cropping led to the accumulation of organic acids and phenolic acids secreted by roots, promoting the growth of pathogenic microorganisms, and further affecting the structure of the rhizospheric soil microbial community (Wu et al., 2019). In this study, we found that the content of fungus changed as described above after continuous cropping of sweet potato, but the specific cause was still uncertain. We infer that continuous cropping of sweet potato leads to changes of the rhizospheric soil fungi living environment. For example, the accumulation of root exudation makes the soil pH and organic matter change to some extent, thus leading to the increase of fungi suitable for such growing conditions and the decrease of those unsuitable. Therefore, the soil physical and chemical properties, soil enzyme activity, root exudates and other factors related to sweet potato continuous cropping, as well as their influences on rhizospheric soil microbial community structure need to be further investigated, so as to provide a more comprehensive basis for prevention and control of continuous cropping-induced sweet potato diseases, reduced pesticide use, and improvement of quality and yield of sweet potato.

## Conclusion

In conclusion, continuous cropping of sweet potato led to the most dominant fungi phylum Ascomycota decreased significantly and the fungi diversity and richness in rhizospheric soil of sweet potato increased significantly. In addition, continuous cropping altered the fungal community structure of the sweet potato rhizospheric soil, such that the content of beneficial fungi decreased, while that of harmful fungi increased, thereby increasing soil-borne diseases and reducing the yield and quality of sweet potato. Furthermore, these effects were different for different sweet potato varieties. Thus, during actual production, attention should be paid to maintain the stability of sweet potato rhizospheric soil microecology through rotation or application of microbial fertilizers and soil amendments to alleviate continuous cropping obstacles.

## Data Availability Statement

The complete data sets generated in our study have been deposited in the NCBI Sequence Read Archive database under accession number SRP215026.

## Author Contributions

ZM, LL, and ZJG designed this experiment. ZL, CL, XW, QT, WJ, and JH executed the experiment. ZYG, MH, and YH finished the manuscript.

### Conflict of Interest

The authors declare that the research was conducted in the absence of any commercial or financial relationships that could be construed as a potential conflict of interest.

## Footnotes

^1^http://rdp.cme.msu.edu/
